# Supplementary material for: Predicting host range expansion in parasitic mites using a global mammalian-acarine dataset
Source: Nat Commun. 2024 Jun 26;15:5431. doi: 10.1038/s41467-024-49515-3 (PMC11208579; doi:10.1038/s41467-024-49515-3)
Supplement: Supplementary file 1 — Supplementary Information [file 41467_2024_49515_MOESM1_ESM.pdf]

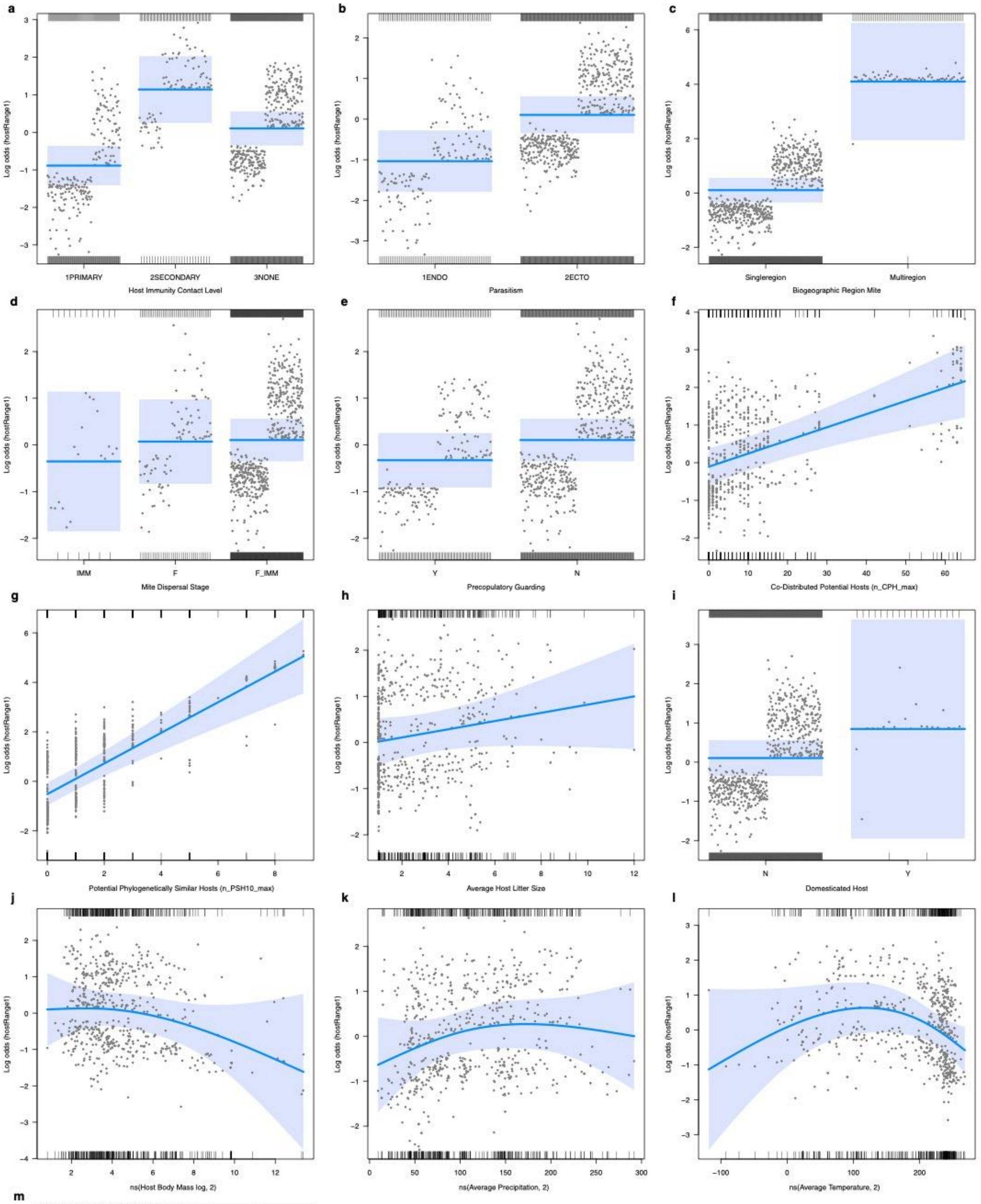


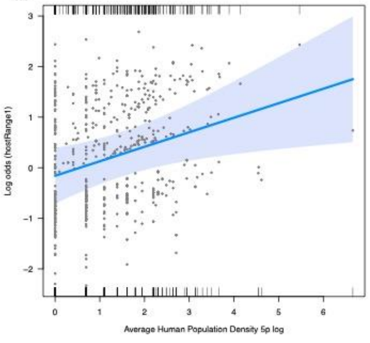


**Supplementary Fig. 1. Spline regression model predicting the probability of becoming multi-host**. **a-m**, Relationships between the outcome (*multihost*) and each of the 13 predictor variables while the other explanatory variables held constant; for each plot, 95% confidence bands (Wald confidence) and partial residuals are shown as both scatterplots and rugs (single-host bottom, multi-host top); ns(var, 2) denotes a two-

degree-of-freedom natural spline. Source data are provided as a Source Data file.

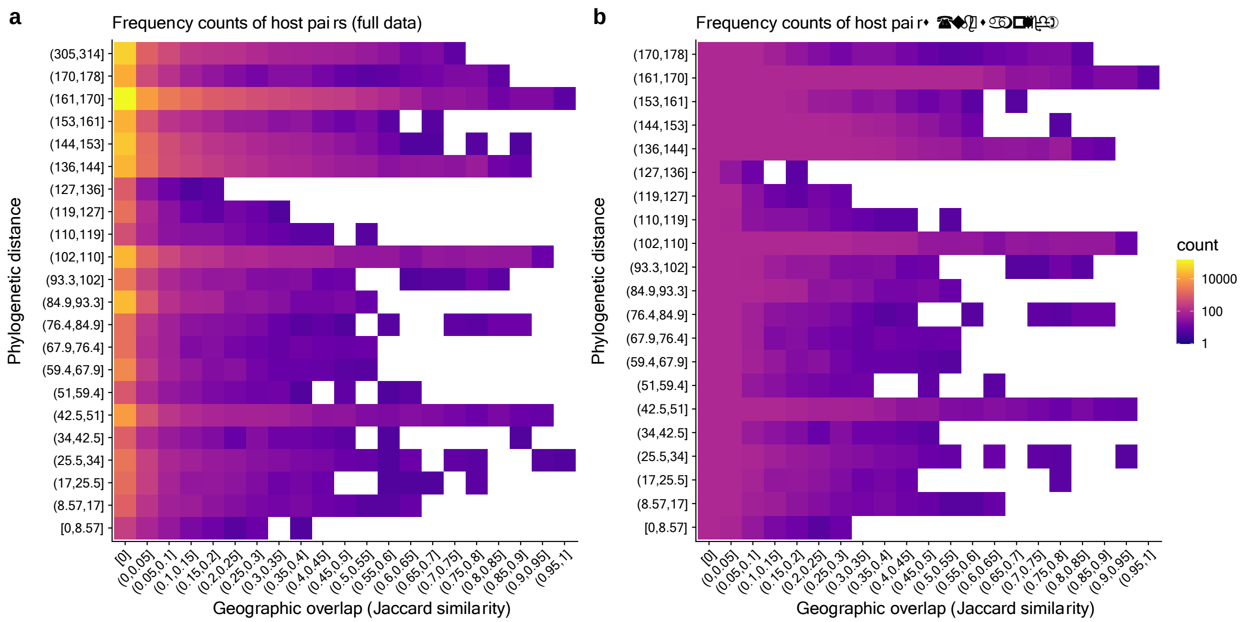


**Supplementary Fig. 2. Frequency counts of host pairs with the combination of value ranges of their geographic overlap and phylogenetic distance**. Geographic overlap was discretized into 20 bins with a step size of 0.05, while phylogenetic distance was discretized into 22 bins. Colors that range from purple to yellow represent log10-scale increase in frequency counts. **a**, full data and **b**, subsampled data used in the logistic regression model to reduce the skewness of variable combinations. Source data are provided as a Source Data file.

**Supplementary Table 1. Summary of logistic regression model predicting mite-sharing probability among host pairs** (see Eq. 2). Independent variables: PD, phylogenetic host distance; GO, geographic overlap (see Eq. 1). The significance of estimated coefficients is indicated using two-sided z-score tests. ns(var,2)=natural spline with 2 degrees of freedom; SE = standard error; z value = ratio of the estimated coefficient to its standard error; Pr(>|z|) = probability of observing a z value as extreme or more extreme than the observed value assuming that the coefficient is zero (null hypothesis); a p-value less than 0.05 indicates that the null hypothesis can be rejected; significance (sign.) codes: *** p<0.001; ** p<0.01; * p<0.05, .<0.1.

| **Coefficients** | **Estimate** | **SE** | **z value** | **Pr(>\|z\|)** | **sign** |
|---|---|---|---|---|---|
| (Intercept) | 0.487 | 0.078 | 6.237 | 4.45E-10 | *** |
| ns(PD, 2)1 | -8.009 | 0.250 | -32.069 | <2.00E-16 | *** |
| ns(PD, 2)2 | -2.119 | 0.211 | -10.058 | <2.00E-16 | *** |
| GO | 1.528 | 0.282 | 5.417 | 6.05E-08 | *** |
| GO:PD | -0.006 | 0.004 | -1.716 | 0.0862 | . |

**Supplementary Table 2. Sensitivity analysis with four species-level taxa of *Opsonyssus*.** *O. brutsaerti indica*, *O. pseudoindicus*, *O. eidoloni*, *O. pteropodi* were coded as endoparasitic. Coefficient estimates and standard errors are given for the best model. This model was constructed following the same methodology as our preferred model for the main analysis (i.e., generalized linear model with splines, applied to a down-sampled dataset). The significance of estimated coefficients is indicated using two-sided z-score tests. CI = confidence interval, lower (LL) and upper limit (UL); SE = standard error; Exp(B) = odds ratios for the predictors (exponentiated model coefficients); z value = ratio of the estimated coefficient to its standard error (absolute value represents the variable importance in the model); Pr = probability of observing a z value (two-sided) as extreme or more extreme than the observed value assuming that the coefficient is zero (null hypothesis); a p-value less than 0.05 indicates that the null hypothesis can be rejected; significance (sign.) codes: *** p<0.001; ** p<0.01; * p<0.05, .<0.1.

| Coefficients | Estimate | CI.LL | CI.LB | SE | Exp(B) | z value | Pr(>\|z\|) | sign |
|---|---|---|---|---|---|---|---|---|
| (Intercept) | -6.006 | -9.161 | -3.339 | 1.468 | 0.002 | -4.092 | 4.28E-05 | *** |
| ImmuneResponse2SECONDARY | 2.051 | 1.130 | 2.962 | 0.468 | 7.775 | 4.381 | 1.18E-05 | *** |
| ImmuneResponse3NONE | 0.989 | 0.431 | 1.564 | 0.289 | 2.689 | 3.427 | 6e-04 | *** |
| Parasitism2ECTO | 1.187 | 0.469 | 1.838 | 0.357 | 3.279 | 3.330 | 9e-04 | *** |
| BioRegCoding3Multiregion | 3.998 | 2.293 | 6.953 | 1.086 | 54.464 | 3.680 | 2e-04 | *** |
| MiteDispersalStageF | 0.226 | -1.222 | 2.063 | 0.828 | 1.254 | 0.273 | 0.7847 | |
| MiteDispersalStageF_IMM | 0.248 | -0.995 | 1.913 | 0.736 | 1.281 | 0.337 | 0.7361 | |
| PrecopulatorGuardingN | 0.442 | -0.178 | 1.049 | 0.313 | 1.555 | 1.411 | 0.1583 | |
| n_CPH_max | 0.035 | 0.019 | 0.051 | 0.008 | 1.036 | 4.277 | 1.90E-05 | *** |
| n_PSH10_max | 0.619 | 0.458 | 0.798 | 0.087 | 1.856 | 7.134 | 9.74E-13 | *** |
| avg_HostLitterSize | 0.090 | -0.032 | 0.210 | 0.061 | 1.094 | 1.456 | 0.1454 | |
| hostDomY | 0.769 | -1.804 | 3.726 | 1.400 | 2.158 | 0.549 | 0.5827 | |
| ns(avg_HostBodyMass_g_log, 2)1 | -0.867 | -2.826 | 1.125 | 1.006 | 0.420 | -0.862 | 0.3888 | |
| ns(avg_HostBodyMass_g_log, 2)2 | -1.768 | -3.945 | 0.250 | 1.065 | 0.171 | -1.660 | 0.097 | . |
| ns(avg_prec, 2)1 | 1.623 | -0.654 | 3.927 | 1.167 | 5.066 | 1.390 | 0.1645 | |
| ns(avg_prec, 2)2 | 0.191 | -1.099 | 1.460 | 0.650 | 1.211 | 0.294 | 0.7686 | |
| ns(avg_temp, 2)1 | 2.848 | -1.345 | 7.433 | 2.244 | 17.252 | 1.269 | 0.2043 | |
| ns(avg_temp, 2)2 | -1.288 | -2.466 | -0.099 | 0.604 | 0.276 | -2.135 | 0.0328 | * |
| avg_HumanPopDen_5p_n_per_km2_log | 0.289 | 0.062 | 0.513 | 0.114 | 1.335 | 2.529 | 0.0115 | * |

**Model metrics**: Accuracy: 0.7506, 95% CI: (0.7064, 0.7912), No Information Rate: 0.7102, P-Value [Acc > NIR]: 0.03671, Kappa: 0.4313, Mcnemar's Test P-Value: 0.01117. 'Positive' Class: 1 (*multihost*): Sensitivity: 0.6803, Specificity: 0.7793, Pos Pred Value: 0.5570, Neg Pred Value: 0.8566, Precision: 0.5570, Recall: 0.6803, F1: 0.6125, Prevalence: 0.2898, Detection Rate: 0.1971, Detection Prevalence: 0.3539, Balanced Accuracy: 0.7298, Precision-Recall AUC: 0.6991111, ROC AUC: 0.7983716.
