# Supplementary material for: Predicting host range expansion in parasitic mites using a global mammalian-acarine dataset
Source: Nat Commun. 2024 Jun 26;15:5431. doi: 10.1038/s41467-024-49515-3 (PMC11208579; doi:10.1038/s41467-024-49515-3)
Supplement: Supplementary file 3 — Description of Additional Supplementary Files [file 41467_2024_49515_MOESM3_ESM.pdf]

Description of Additional Supplementary Files

Supplementary Data 1. High epidemic risk-group mites identified by our preferred model (generalized linear model with splines). Single-hosts predicted as multi-host at threshold 0.7. Supplementary_Data1_Preferred_Model_Forecast_Singlehost_predicted_as_Multihost_at_Threshold_0.7.xlsx

Supplementary Data 2. High epidemic risk-group mites identified by our alternative PU model. Single-hosts predicted as multi-host at threshold 0.7. Supplementary_Data2_PU_Model_Forecast_SingleHost_predicted_as_Multihost_at_Threshold_0.7.xlsx

Supplementary Data 3. Full host-parasite database. Supplementary_Data3_mammal_mite_db.v12-flattened.xlsx

Supplementary Data 4. Analysis dataset. Supplementary_Data4_Mammal.parasites.analysis.mite_data.v25.xlsx
