# Supplementary material for: Predicting host range expansion in parasitic mites using a global mammalian-acarine dataset
Source: Nat Commun. 2024 Jun 26;15:5431. doi: 10.1038/s41467-024-49515-3 (PMC11208579; doi:10.1038/s41467-024-49515-3)
Supplement: Supplementary file 8 — Reporting Summary [file 41467_2024_49515_MOESM8_ESM.pdf]

nature portfolio

Corresponding author(s): Pavel Klimov

Last updated by author(s): 05/07/2024

Reporting Summary

Nature Portfolio wishes to improve the reproducibility of the work that we publish. This form provides structure for consistency and transparency in reporting. For further information on Nature Portfolio policies, see our Editorial Policies and the Editorial Policy Checklist.

Statistics

For all statistical analyses, confirm that the following items are present in the figure legend, table legend, main text, or Methods section.

n/a	Confirmed	
☐	☒	The exact sample size (*n*) for each experimental group/condition, given as a discrete number and unit of measurement
☐	☒	A statement on whether measurements were taken from distinct samples or whether the same sample was measured repeatedly
☐	☒	The statistical test(s) used AND whether they are one- or two-sided<br>*Only common tests should be described solely by name; describe more complex techniques in the Methods section.*
☐	☒	A description of all covariates tested
☐	☒	A description of any assumptions or corrections, such as tests of normality and adjustment for multiple comparisons
☐	☒	A full description of the statistical parameters including central tendency (e.g. means) or other basic estimates (e.g. regression coefficient) AND variation (e.g. standard deviation) or associated estimates of uncertainty (e.g. confidence intervals)
☐	☒	For null hypothesis testing, the test statistic (e.g. *F*, *t*, *r*) with confidence intervals, effect sizes, degrees of freedom and *P* value noted<br>*Give P values as exact values whenever suitable.*
☒	☐	For Bayesian analysis, information on the choice of priors and Markov chain Monte Carlo settings
☒	☐	For hierarchical and complex designs, identification of the appropriate level for tests and full reporting of outcomes
☐	☒	Estimates of effect sizes (e.g. Cohen's *d*, Pearson's *r*), indicating how they were calculated

Our web collection on statistics for biologists contains articles on many of the points above.

Software and code

Policy information about availability of computer code

Data collection	Literature search, Google Scholar and the Zoological Record database. Data were transcribed from actual literature references.
Data analysis	R v4.2.2 and 4.3.1; R packages: caret 6.0-94, splines 4.2.2 and 4.3.1; custom R code: DOI: 10.5281/zenodo.11130647

For manuscripts utilizing custom algorithms or software that are central to the research but not yet described in published literature, software must be made available to editors and reviewers. We strongly encourage code deposition in a community repository (e.g. GitHub). See the Nature Portfolio guidelines for submitting code & software for further information.

Data

Policy information about availability of data

All manuscripts must include a data availability statement. This statement should provide the following information, where applicable:

- Accession codes, unique identifiers, or web links for publicly available datasets
- A description of any restrictions on data availability
- For clinical datasets or third party data, please ensure that the statement adheres to our policy

All data used in this study were provided in Supplementary Data or on Zenodo DOI: 10.5281/zenodo.11130647.

Research involving human participants, their data, or biological material

Policy information about studies with human participants or human data. See also policy information about sex, gender (identity/presentation), and sexual orientation and race, ethnicity and racism.

Reporting on sex and gender	N/A
Reporting on race, ethnicity, or other socially relevant groupings	N/A
Population characteristics	N/A
Recruitment	N/A
Ethics oversight	N/A

Note that full information on the approval of the study protocol must also be provided in the manuscript.

Field-specific reporting

Please select the one below that is the best fit for your research. If you are not sure, read the appropriate sections before making your selection.

☐ Life sciences ☐ Behavioural & social sciences ☒ Ecological, evolutionary & environmental sciences

For a reference copy of the document with all sections, see nature.com/documents/nr-reporting-summary-flat.pdf

Ecological, evolutionary & environmental sciences study design

All studies must disclose on these points even when the disclosure is negative.

Study description	To predict mite host range (single-host vs multi-host), we collected the following 14 predictor variables from the literature: (i) mite-related: Host Immunity Contact Level, Chelicerae, Parasitism, Mite Bioregion, Mite Dispersal Stage, Precopulatory Guarding; (ii) host-related: Co-Distributed Potential Hosts, Potential Phylogenetically Similar Hosts, Average Host Litter Size, Domesticated Host, Average Host Body Mass log, (iii) climatic: Average Precipitation, Average Temperature, and (iv) habitat disturbance: Average Human Population Density log. These variables were numeric: Co-Distributed Potential Hosts, Potential Phylogenetically Similar Hosts, Average Host Litter Size, Host Body Mass, Average Precipitation, Average Temperature, Average Human Population Density. The remaining variables were factors.
Research sample	Using the literature, we compiled a taxonomic database of parasitic acariform mites, including the following information: (1) valid mite name, authority, and year; (2) unique host records per mite species; (3) mite taxonomy (family, parvorder); (4) biogeographic region; and (5) credibility of host record (see below). Our database has 3,489 unique host-parasite records representing 1,998 mite and 1,486 mammalian species (Supplementary Data 3). Based on interviewing authors about their sampling procedures and the repeatability of a particular association, we marked 113 records as potential sample cross-contamination in the field, laboratory or museum (low credibility). After quality control (e.g., missing data, uncertainty in host identification, low credibility records) and synchronizing the host taxonomy with the latest source, the Mammal Diversity Database (MDD) v1.10, our dataset had 3,350 unique host-parasite records representing 1,984 mite species (22 families, 2 parvorders) and 1,432 mammal species (118 families, 22 orders). A host name lookup table was created to retrieve data from four external sources, all using different host taxonomies: host classification, domestication status, and biogeographic region (MDD v1.10); host phylogeny; host traits and environmental data (PanTHERIA); shape files describing spatial distribution of mammalian hosts (MDD v1.2); and Google Scholar publication count per mite species accessed on May 20 2023.
Sampling strategy	We used exhaustive sampling (all unique host-parasite records known to date).
Data collection	Mite data were collected from the literature by PBK. Host, climatic, and environmental data were collected from public databases by PBK and QH.
Timing and spatial scale	1758-2022
Data exclusions	Based on interviewing authors about their sampling procedures and the repeatability of a particular association, we excluded 113 records as potential sample cross-contamination in the field, laboratory or museum.
Reproducibility	All the codes used for generating the figures and tables are provided on Zenodo DOI: 10.5281/zenodo.11130647
Randomization	We subsampled (up- and down-sampling) and resampled (5-fold cross-validations, 5 repeats) our train dataset, and selected the best model based on the their performances on an independent test dataset.
Blinding	Blinding is not relevant to our study. We analyse known attributes of species (not subjects)

Did the study involve field work? ☐ Yes ☒ No

Reporting for specific materials, systems and methods

We require information from authors about some types of materials, experimental systems and methods used in many studies. Here, indicate whether each material, system or method listed is relevant to your study. If you are not sure if a list item applies to your research, read the appropriate section before selecting a response.

Materials & experimental systems

n/a	Involved in the study	
☒	☐	Antibodies
☒	☐	Eukaryotic cell lines
☒	☐	Palaeontology and archaeology
☒	☐	Animals and other organisms
☒	☐	Clinical data
☒	☐	Dual use research of concern
☒	☐	Plants

Methods

n/a	Involved in the study	
☒	☐	ChIP-seq
☒	☐	Flow cytometry
☒	☐	MRI-based neuroimaging
